# Evaluating the Diagnostic Performance of Symptom Checkers: Clinical Vignette Study

**DOI:** 10.2196/46875

**Published:** 2024-04-29

**Authors:** Mohammad Hammoud, Shahd Douglas, Mohamad Darmach, Sara Alawneh, Swapnendu Sanyal, Youssef Kanbour

**Affiliations:** 1 Avey Inc Doha Qatar

**Keywords:** digital health, symptom checker, artificial intelligence, AI, patient-centered care, eHealth apps, eHealth

## Abstract

**Background:**

Medical self-diagnostic tools (or symptom checkers) are becoming an integral part of digital health and our daily lives, whereby patients are increasingly using them to identify the underlying causes of their symptoms. As such, it is essential to rigorously investigate and comprehensively report the diagnostic performance of symptom checkers using standard clinical and scientific approaches.

**Objective:**

This study aims to evaluate and report the accuracies of a few known and new symptom checkers using a standard and transparent methodology, which allows the scientific community to cross-validate and reproduce the reported results, a step much needed in health informatics.

**Methods:**

We propose a 4-stage experimentation methodology that capitalizes on the standard clinical vignette approach to evaluate 6 symptom checkers. To this end, we developed and peer-reviewed 400 vignettes, each approved by at least 5 out of 7 independent and experienced primary care physicians. To establish a frame of reference and interpret the results of symptom checkers accordingly, we further compared the best-performing symptom checker against 3 primary care physicians with an average experience of 16.6 (SD 9.42) years. To measure accuracy, we used 7 standard metrics, including M1 as a measure of a symptom checker’s or a physician’s ability to return a vignette’s main diagnosis at the top of their differential list, *F*_1_-score as a trade-off measure between recall and precision, and Normalized Discounted Cumulative Gain (NDCG) as a measure of a differential list’s ranking quality, among others.

**Results:**

The diagnostic accuracies of the 6 tested symptom checkers vary significantly. For instance, the differences in the M1, *F*_1_-score, and NDCG results between the best-performing and worst-performing symptom checkers or ranges were 65.3%, 39.2%, and 74.2%, respectively. The same was observed among the participating human physicians, whereby the M1, *F*_1_-score, and NDCG ranges were 22.8%, 15.3%, and 21.3%, respectively. When compared against each other, physicians outperformed the best-performing symptom checker by an average of 1.2% using *F*_1_-score, whereas the best-performing symptom checker outperformed physicians by averages of 10.2% and 25.1% using M1 and NDCG, respectively.

**Conclusions:**

The performance variation between symptom checkers is substantial, suggesting that symptom checkers cannot be treated as a single entity. On a different note, the best-performing symptom checker was an artificial intelligence (AI)–based one, shedding light on the promise of AI in improving the diagnostic capabilities of symptom checkers, especially as AI keeps advancing exponentially.

## Introduction

### Background

Digital health has become ubiquitous. Every day, millions of people turn to the internet for health information and treatment advice [1,2]. For instance, in Australia, approximately 80% of people search the internet for health information and approximately 40% seek web-based guidance for self-treatment [3,4]. In the United States, approximately two-thirds of adults search the web for health information and one-third use it for self-diagnosis, trying to singlehandedly understand the underlying causes of their health symptoms [5]. A recent study showed that half of the patients investigated their symptoms on search engines before visiting emergency rooms [6,7].

Although search engines such as Google and Bing are exceptional tools for educating people on almost any matter, they may facilitate misdiagnosis and induce serious risks [5]. This is because searching the web entails sifting through a great deal of information, stemming from all kinds of sources, and making personal medical judgments, correlations, and deductions accordingly. Some governments have even launched “Don’t Google It” advertising campaigns to raise public awareness of the risks of assessing one’s health using search engines [8,9]. The reality is that search engines are not medical diagnostic tools and laymen are not usually equipped to leverage them for self-diagnosis.

In contrast to search engines, symptom checkers are patient-facing medical diagnostic tools that emulate clinical reasoning, especially if they use artificial intelligence (AI) [4,10]. They are trained to make medical expert–like judgments on behalf of patients. More precisely, a patient can start a consultation session with a symptom checker by inputting a chief complaint (in terms of ≥1 symptoms). Afterward, the symptom checker asks several questions to the patient and collects answers from them. Finally, it generates a differential diagnosis (ie, a ranked list of potential diseases) that explains the causes of the patient’s symptoms.

Symptom checkers are increasingly becoming an integral part of digital health, with >15 million people using them on a monthly basis [11], a number that is expected to continue to grow [12]. A United Kingdom–based study [13] that engaged 1071 patients found that >70% of individuals aged between 18 and 39 years would use a symptom checker. A recent study examining a specific symptom checker found that >80% of patients perceived it to be useful and >90% indicated that they would use it again [14]. Various credible health care institutions and entities such as the UK National Health Service [15] and the government of Australia [16] have officially adopted symptom checkers for self-diagnosis and referrals.

Symptom checkers are inherently scalable (ie, they can assess millions of people instantly and concurrently) and universally available. In addition, they promise to provide patients with necessary high-quality, evidence-based information [17]; reduce unnecessary medical visits [18-21]; alleviate the pressure on health care systems [22]; improve accessibility to timely diagnosis [18]; and guide patients to the most appropriate care pathways [12], to mention just a few.

Nevertheless, the utility and promise of symptom checkers cannot be materialized if they are not proven to be accurate [10]. To elaborate, a recent study has shown that most patients (>76%) use symptom checkers solely for self-diagnosis [14]. As such, if symptom checkers are not meticulously engineered and rigorously evaluated on their diagnostic capabilities, they may put patients at risk [23-25].

This study investigates the diagnostic performance of symptom checkers by measuring the accuracies of a few popular symptom checkers and a new AI-based symptom checker. In addition, it compares the accuracy of the best-performing symptom checker against that of a panel of experienced physicians to put things in perspective and interpret results accordingly.

### Evaluation Methodology

To evaluate symptom checkers, we propose a scientific methodology that capitalizes on the standard clinical vignette approach [26] (Multimedia Appendix 1 provides additional information on how our methodology aligns with the recommended requirements of this approach [4,7,12,26-39]). Delivering on this methodology, we compiled 400 vignettes and peer reviewed them with 7 external physicians using a supermajority voting scheme. To the best of our knowledge, this yielded the largest benchmark vignette suite in the domain thus far. Furthermore, we defined and used 7 standard accuracy metrics, one of which measures for the first time, the ranking qualities of the differential diagnoses of symptom checkers and physicians.

Subsequently, we leveraged the peer-reviewed benchmark vignette suite and accuracy metrics to investigate the performance of a new AI-based symptom checker named Avey [40] and 5 popular symptom checkers named Ada [41], K Health [42], Buoy [43], Babylon [44], and WebMD [45]. Results demonstrated a significant performance variation between these symptom checkers and the promise of AI in improving their diagnostic capabilities. For example, the best-performing symptom checker, namely Avey, outperformed Ada, K Health, Buoy, Babylon, and WebMD by averages of 24.5%, 142.8%, 159.6%, 2968.1%, and 175.5%, respectively, in listing the vignettes’ main diagnoses at the top of their differentials.

**Figure 1:**
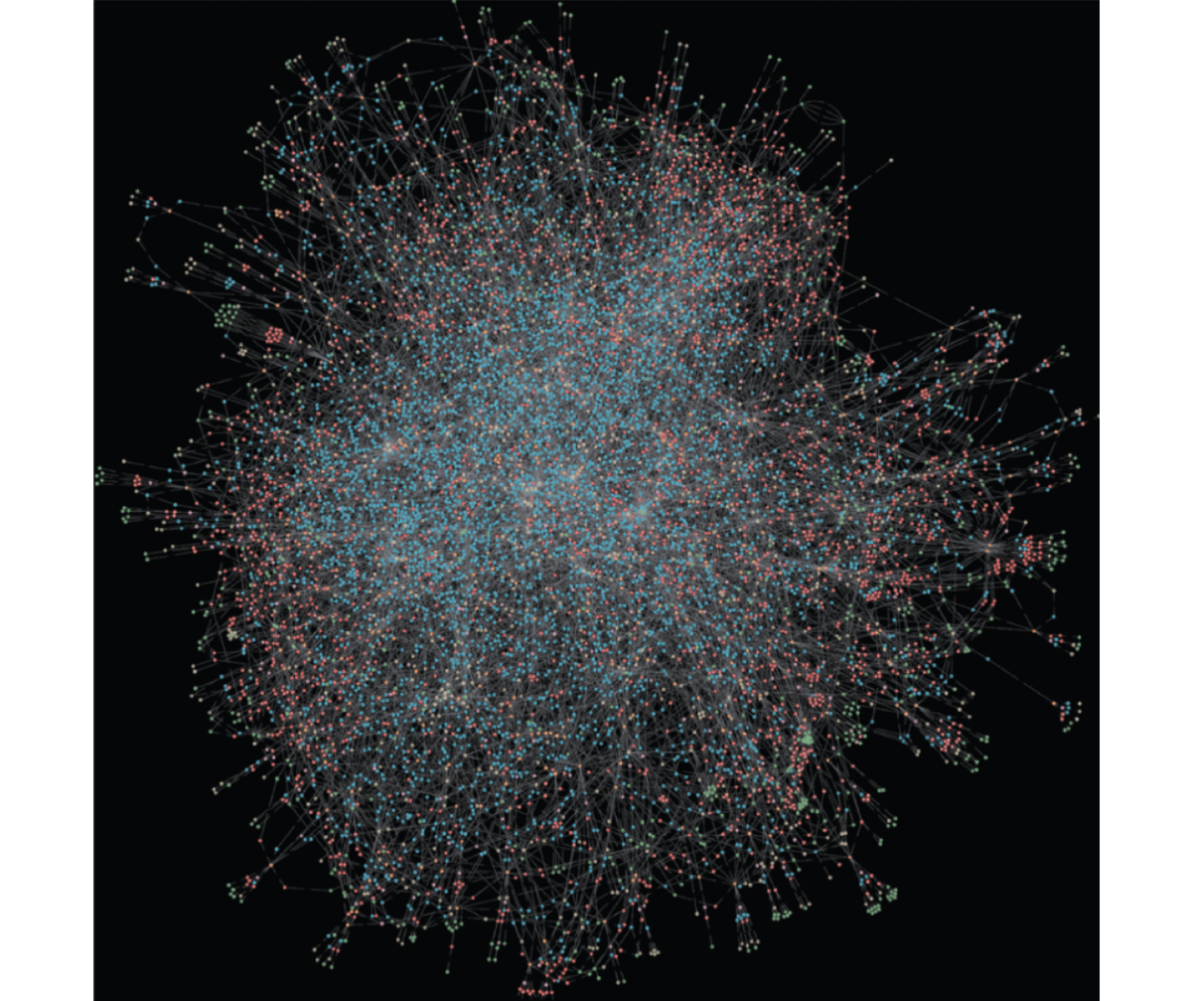
An actual visualization of Avey’s brain (ie, a probabilistic graphical model). At a high level, the nodes (or dots) can be thought of representing diseases, symptoms, etiologies, or features of symptoms or etiologies, whereas the edges (or links) can be thought of as representing conditional independence assumptions and modeling certain features (eg, sensitivities and specificities) needed for clinical reasoning.

Avey claims to use advanced AI technology [40]. In particular, it involves a diagnostic engine that operationalizes a probabilistic graphical model, namely a Bayesian network. Figure 1 demonstrates the model in action, which was built bottom-up over 4 years specifically for medical diagnosis. In addition, the engine uses a recommendation system, which predicts the future impact of every symptom or etiology that has not yet been asked during a patient session with Avey and recommends the one that exhibits the highest impact on the engine’s current diagnostic hypothesis. At the end of the session, a ranking model is used for ranking all the possible diseases for the patient’s case and outputs them as a differential diagnosis.

To put things in perspective, we subsequently compared the performance of Avey against 3 primary care physicians with an average experience of 16.6 years. The results showed that Avey compared favorably to the physicians and slightly outperformed them in some accuracy metrics, including the ability to rank diseases correctly within their generated differential lists.

Finally, to facilitate the reproducibility of the study and support future related studies, we made the peer-reviewed benchmark vignette suite publicly and freely available [27]. In addition, we posted all the results of the symptom checkers and physicians in the Benchmark Vignette Suite [27] to establish a standard of full transparency and allow researchers to cross-validate the results, a step much needed in health informatics [46].

## Methods

### Stages

#### Overview

Building on prior related work [4,5,11,12,26,28,29], we adopted a clinical vignette approach to measure the performance of symptom checkers. A seminal work at Harvard Medical School has established the value of this approach in validating the accuracies of symptom checkers [11,29], especially because it has been also used as a common approach to test physicians on their diagnostic capabilities [29].

**Figure 2:**
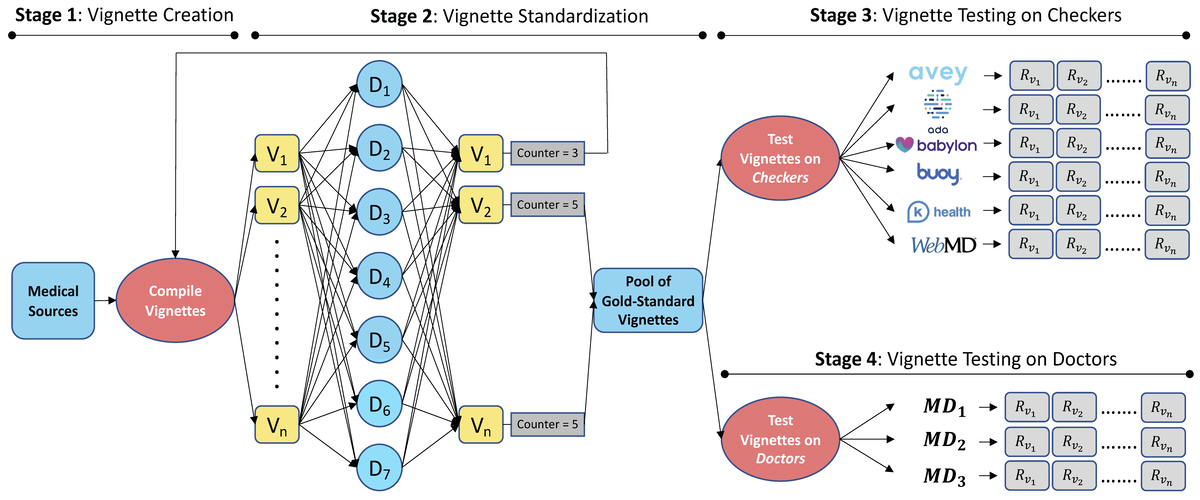
Our 4-stage experimentation methodology (V_i_=vignette i, assuming n vignettes and 1≤i≤n; D_j_=doctor j, assuming 7 doctors and 1≤j≤7; MD_k_=medical doctor k, assuming 3 doctors and 1≤k≤3; R_i_=result of vignette V_i_ as generated by a checker or a medical doctor [MD]). In the “vignette creation” stage, the vignettes are compiled from reputable medical sources by an internal team of MDs. In the “vignette standardization” stage, the vignettes are reviewed and approved by a panel of experienced and independent physicians. In the “vignette testing on symptom checkers” stage, the vignettes are tested on symptom checkers by a different panel of experienced and independent physicians. In the “vignette testing on doctors” stage, the vignettes are tested on a yet different panel of experienced and independent physicians.

To this end, we defined our experimentation methodology in terms of 4 stages, namely *vignette creation*, *vignette standardization*, *vignette testing on symptom checkers*, and *vignette testing on doctors*. The 4 stages are illustrated in Figure 2.

#### Stage 1: Vignette Creation Stage

In this stage, an internal team of 3 physicians (akin to the study by Gilbert et al [28]) compiled a set of vignettes from October 10, 2021, to November 29, 2021. All the vignettes were drawn from reputable medical websites and training material for health care professionals, including the United States Medical Licensing Examination, Step 2 CK, Membership of the Royal Colleges of Physicians Part 1 Self-Assessment, American Board of Family Medicine, and American Board of Pediatrics, among others [30-37]. In addition, the internal medical team supplemented the vignettes with information that might be “asked” by symptom checkers and physicians in stages 3 and 4. The vignettes involved 14 body systems and encompassed common and less-common conditions relevant to primary care practice (Table 1). They fairly represent real-life or practical cases in which patients might seek primary care advice from physicians or symptom checkers.

The internal medical team constructed each vignette in terms of eight major components: (1) the age and sex of the assumed patient; (2) a maximum of 3 chief complaints; (3) the history of the suggested illness associated with details on the chief complaints and other present and relevant *findings* (a finding is defined as a symptom, a sign, or an etiology, each with a potential attribute); (4) absent findings, including ones that are expected to be solicited by symptom checkers and physicians in stages 3 and 4; (5) basic findings that pertain to physical examinations that can still be exploited by symptom checkers; (6) past medical and surgical history; (7) family history; and (8) the most appropriate main and differential diagnoses.

**Table 1:** The body systems and numbers of common and less-common diseases covered in the compiled vignette suite.

Body system	Vignettes				Covered diseases, % (p^a^/P^b^)
	Weightage in the suite, % (n^c^/N^d^)	Vignettes with common diseases % (m^e^/n) (total: 55.5%, 222/400)	Vignettes with less-common diseases, % (k^f^/n) (total: 44.5%, 178/400)		
Hematology	5.75 (23/400)	8.7 (2/23)	91.3 (21/23)	4.89 (13/266)	
Cardiovascular	11.5 (46/400)	58.7 (27/46)	41.3 (19/46)	11.28 (30/266)	
Neurology	5.5 (22/400)	40.91 (9/22)	59.09 (13/22)	5.26 (14/266)	
Endocrine	20 (5)5 (20/400)	65 (13/20)	35 (7/20)	4.89 (13/266)	
ENT^g^	5.75 (23/400)	69.57 (16/23)	30.43 (7/23)	5.64 (15/266)	
GI^h^	11 (44/400)	47.73 (21/44)	52.27 (23/44)	12.78 (34/266)	
Obstetrics and gynecology	13.5 (54/400)	59.26 (32/54)	40.74 (22/54)	13.16 (35/266)	
Infectious	5.75 (23/400)	26.09(6/23)	73.91 (17/23)	6.39 (17/266)	
Respiratory	9.25 (37/400)	70.27 (26/37)	29.73 (11/37)	7.52 (20/266)	
Orthopedics and rheumatology	8 (32/400)	65.63 (21/32)	34.38 (11/32)	9.4 (25/266)	
Ophthalmology	4.5 (18/400)	83.33 (15/18)	16.67 (3/18)	4.51 (12/266)	
Dermatology	3 (12/400)	75 (9/12)	25 (3/12)	4.51 (12/266)	
Urology	3.5 (14/400)	57.14 (8/14)	42.86 (6/14)	3.01 (8/266)	
Nephrology	8 (32/400)	53.13 (17/32)	46.88 (15/32)	6.77 (18/266)	

^a^p: number of diseases covered in the body system.

^b^P: total number of diseases covered by the N vignettes.

^c^n: number of vignettes for the corresponding body system.

^d^N: total number of vignettes in our suite.

^e^m: count of vignettes covering common diseases of the corresponding body system.

^f^k: count of vignettes covering less-common diseases of the corresponding body system.

^g^ENT: ear, nose, and throat.

^h^GI: gastrointestinal.

#### Stage 2: Vignette Standardization Stage

The output of the vignette creation stage (ie, stage 1) is a set of vignettes that serves as an input to the vignette standardization stage (ie, stage 2). Seven external physicians (as opposed to 3 doctors in the study by Gilbert et al [28]) from 4 specialties, namely family medicine, general medicine, emergency medicine, and internal medicine, with an average experience of 8.4 years were recruited from the professional networks of the authors to review the vignettes in this stage. None of these external doctors had any involvement with the development of any of the symptom checkers considered in this study.

We designed and developed a full-fledged web portal to streamline the process of reviewing and standardizing the vignettes. To elaborate, the portal allows the internal medical team to upload the vignettes to a web page that is shared across the 7 externally recruited doctors. Each doctor can access the vignettes and review them independently, without seeing the reviews of other doctors.

After reviewing a vignette, a doctor can reject or accept it. Upon rejecting a vignette, a doctor can propose changes to improve its quality or clarity. The internal medical team checks the suggested changes, updates the vignette accordingly, and reuploads it to the portal for a new round of peer reviewing by the 7 external doctors. Multiple reviewing rounds can take place before a vignette is rendered gold standard. A vignette becomes the gold standard only if it is accepted by at least 5 out of the 7 (ie, supermajority) external doctors. Once a vignette is standardized, the portal moves it automatically to stages 3 and 4.

Stage 2 started on October 17, 2021, and ended on December 4, 2021. As an outcome, 400 vignettes were produced and standardized. To allow for external validation, we made all the vignettes publicly available [27].

#### Stage 3: Vignette Testing on Symptom Checkers

The output of stage 2 serves as an input to stage 3, namely, vignette testing on symptom checkers. For this sake, we recruited 3 independent primary care physicians under 2 specialties, namely family medicine and general medicine, with an average experience of 4.2 years from the professional networks of the authors. None of these physicians had any involvement with the development of any of the symptom checkers tested in this study. Furthermore, 2 of them were not among the 7 doctors who reviewed the vignettes in stage 2. These doctors were recruited solely to test the gold-standard vignettes on the considered symptom checkers.

The approach of having primary care physicians test symptom checkers has been shown recently to be more reliable than having laypeople do so [28,38,47]. This is because the standardized vignettes act as proxies for patients, whereas testers act as only data extractors from the vignettes and information feeders to the symptom checkers. Consequently, the better the testers are in extracting and feeding data, the more reliable the clinical vignette approach renders. In fact, a symptom checker cannot be judged on its accuracy if the answers to its questions are not in full alignment with the contents of the vignettes.

To this end, physicians are deemed more capable of playing the role of testers than laypeople, especially that AI-based symptom checkers (eg, Ada and Avey, among others) may often ask questions that have no answers in the vignettes, even if the vignettes are quite comprehensive. Clearly, when these questions are asked, laypeople will not be able to answer them properly, impacting thereby the reliability of the clinical vignette approach and the significance of the reported results. In contrast, physicians will judiciously answer these questions in alignment with the vignettes and capably figure out whether the symptom checkers are able to “diagnose” them (ie, produce the correct differential diagnoses in the vignettes). We elaborate further on the rationale behind using physicians as testers in the Strengths and Limitations section.

Besides vignettes, we chose 6 symptom checkers, namely Ada [41], Babylon [44], Buoy [43], K Health [42], WebMD [45], and Avey [40], to evaluate their performance and compare them against each other. Four of these symptom checkers (ie, Ada, Buoy, K Health, and WebMD) were selected because of their superior performance reported in Gilbert et al [28], and 1 (ie, Babylon) was chosen because of its popularity. Avey is a new AI-based symptom checker that is emerging, with >1 million people who have already downloaded it [40]. We tested the gold-standard vignettes on the most up-to-date versions of these symptom checkers that were available on Google Play, App Store, or websites (eg, Buoy) between the dates of November 7, 2021, and January 31, 2022.

The 6 symptom checkers were tested through their normal question-answer flows. As in the study by Gilbert et al [28], each of the external physicians in stage 3 randomly pulled vignettes from the gold-standard pool and tested them on *each* of the 6 symptom checkers (compared to the study by Gilbert et al [28], where 8 doctors tested vignettes on 4 symptom checkers; Figure 2). By the end of stage 3, each physician tested a total of 133 gold-standard vignettes on each symptom checker, except 1 physician who tested 1 extra vignette to exhaust the 400 vignettes. Each physician saved a screenshot of each symptom checker’s output for each vignette to facilitate the results’ verification, extraction, and analysis. We posted all the screenshots on the internet on the internet [27] to establish a standard of full transparency and allow for external cross-validation and study replication.

#### Stage 4: Vignette Testing on Doctors

In this stage, we recruited 3 more independent and experienced primary care physicians with an average experience of 16.6 years (compared with 7 doctors in the study by Gilbert et al [28], with an average experience of 11.2 years) from the professional networks of the authors. One of those physicians is a family medicine doctor with >30 years of experience. The other 2 are also family medicine doctors, each with >10 years of experience. None of these physicians had any involvement with the development of any of the tested symptom checkers. Furthermore, none of them was among the 7 or 3 doctors of stages 2 or 3, respectively, and they were all only recruited to pursue stage 4.

The sole aim of stage 4 is to compare the accuracy of the winning symptom checker against that of experienced primary care physicians. Hence, similar to the study by Semigran et al [11], we concealed the main and differential diagnoses of the 400 gold-standard vignettes from the 3 recruited doctors and exposed the remaining information through our web portal. The doctors were granted access to the portal and asked to provide their main and differential diagnoses for each vignette without checking any reference, mimicking as closely as possible the way they conduct real-world sessions live with patients. As an outcome, each vignette was “diagnosed” by each of the 3 doctors. The results of the doctors were posted to allow for external cross-validation [27].

Finally, we note that different symptom checkers and doctors can refer to the same disease differently. As such, we considered an output disease by a symptom checker (in stage 3) or a doctor (in stage 4) as a reasonable match to a disease in the gold-standard vignette if it was an alternative name, an umbrella name, or a directly related disease.

### Accuracy Metrics

To evaluate the performance of symptom checkers and doctors in stages 3 and 4, we used 7 standard accuracy metrics. As in the study by Gilbert et al [28] and United States Medical Licensing Examination [48], for every tested gold-standard vignette, we used the matching-1 (M1), matching-3 (M3), and matching-5 (M5) criteria to measure if a symptom checker or a doctor is able to output the vignette’s main diagnosis at the top (ie, *M*1), among the first 3 diseases (ie, *M*3), or among the first 5 diseases (ie, *M*5) of their differential list. For each symptom checker and doctor, we report the percentages of vignettes that fulfill *M*1, *M*3, and *M*5. The mathematical definitions of *M*1, *M*3, and *M*5 are given in Table 2.

Besides, as in the studies by Gilbert et al [28], Baker et al [38], and Kannan et al [49], for each tested gold-standard vignette, we used recall (or sensitivity in medical parlance) as a measure of the percentage of relevant diseases that are returned in the symptom checker’s or doctor’s differential list. Moreover, we used precision as a measure of the percentage of diseases in the symptom checker’s or doctor’s differential list that are relevant. For each symptom checker and doctor, we report the average recall and average precision (see Table 2 for their mathematical definitions) across all vignettes.

Typically, there is a trade-off between recall and precision (the higher the recall, the lower the precision, and vice versa). Thus, in accordance with the standard practice in computer science, we further used the *F*_1_-measure that combines the trade-off between recall and precision in one easily interpretable score. The mathematical definition of the *F*_1_-measure is provided in Table 2. The higher the *F*_1_-measure of a symptom checker or a doctor, the better.

**Table 2:** The descriptions and mathematical definitions of the 7 accuracy metrics used in this study.

Metric	Description	Mathematical definition
M1%	The percentage of vignettes where the gold standard main diagnosis is returned at the top of a symptom checker’s or a doctor’s differential list	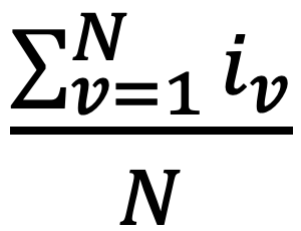 , where *N* is the number of vignettes and *i*_*v*_ is 1 if the symptom checker or doctor returns the gold standard main diagnosis within vignette *v* at the top of their differential list; and 0 otherwise
M3%	The percentage of vignettes where the gold standard main diagnosis is returned among the first 3 diseases of a symptom checker’s or a doctor’s differential list	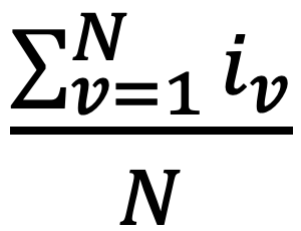 , where *N* is the number of vignettes and *i*_*v*_ is 1 if the symptom checker or doctor returns the gold standard main diagnosis within vignette *v* among the top 3 diseases of their differential list; and 0 otherwise
M5%	The percentage of vignettes where the gold standard main diagnosis is returned among the first 5 diseases of a symptom checker’s or a doctor’s differential list	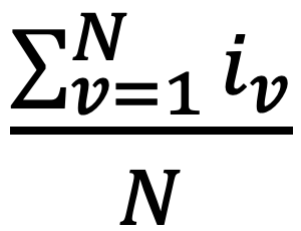 , where *N* is the number of vignettes and *i*_*v*_ is 1 if the symptom checker or doctor returns the gold standard main diagnosis within vignette *v* among the top 5 diseases of their differential list; and 0 otherwise
Average recall	Recall is the proportion of diseases that are in the gold standard differential list and are generated by a symptom checker or a doctor. The average recall is taken across all vignettes for each symptom checker and doctor	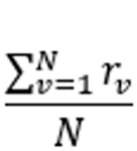 , where *N* is the number of vignettes and 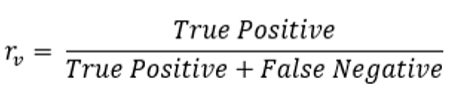 of the symptom checker or doctor for vignette *v*
Average precision	Precision is the proportion of diseases in the symptom checker’s or doctor’s differential list that are also in the gold standard differential list. The average precision is taken across all vignettes for each symptom checker and doctor	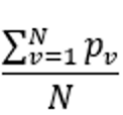 , where *N* is the number of vignettes and 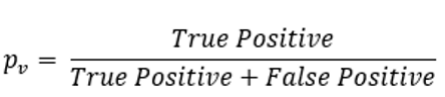 of the symptom checker or doctor for vignette *v*
Average *F*_1_-measure	*F*_1_-measure captures the trade-off between precision and recall. The average *F*_1_-measure is taken across all vignettes for each symptom checker and doctor	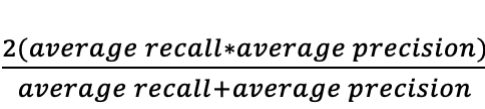 , where *average recall* and *average precision* are as defined at column 3 in rows 4 and 5 above, respectively
Average NDCG^a^	NDCG is a measure of ranking quality. The average NDCG is taken across all vignettes for each symptom checker and doctor	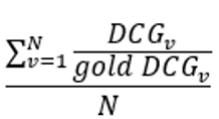 , assuming *N* vignettes, *n* number of diseases in a gold standard vignette *v*, and *relevance*_*i*_ for the disease at position 𝑖 in *v*’s differential list 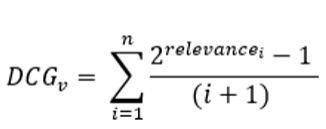 , which is computed over the differential list of a doctor or a symptom checker for *v*. *Gold DCG*_*v*_ is defined exactly as *DCG*_*v*_, but is computed over the gold standard differential list of v

^a^NDCG: Normalized Discounted Cumulative Gain.

Finally, we measured the ranking qualities of each symptom checker and doctor using the Normalized Discounted Cumulative Gain (NDCG) [50] metric that is widely used in practice [51]. To begin with, each disease at position in the differential list of a gold-standard vignette is assigned . The higher the rank of a disease in the differential list, the higher the relevance of that disease to the correct diagnosis (eg, if a gold-standard differential has 2 diseases D1 and D2 in this order, they will be assigned relevancies 2 and 1, respectively). Next, Discounted Cumulative Gain (DCG) is defined mathematically as 
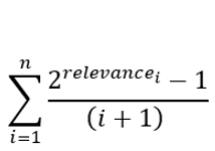
, assuming diseases in a vignette’s differential list (Table 2). As such, DCG penalizes a symptom checker or a doctor if they rank a disease lower in their output differential list than the gold-standard list. Capitalizing on DCG, NDCG is the ratio of a symptom checker’s or a doctor’s DCG divided by the corresponding gold-standard DCG. Table 2 provides the mathematical definition of NDCG.

### Ethical Considerations

No patients (whether as *subjects* or *testers*) were involved in any part of this study, but rather vignettes that acted as proxies for patients during testing with symptom checkers and physicians. As such, the vignettes are the subjects in this study and not humans. In addition, doctors were not subjects in stage 4 of the study (or any stage as a matter of fact), but rather the vignettes themselves. When the subjects are not humans, no institutional review board approval is typically required as per the guidelines of the United States Office for Human Research Protections [52]. This closely aligns with many of the related studies that use the clinical vignette approach [12,28,29,38,53,54], whereby none of them (to the best of our knowledge) has obtained an institutional review board approval to conduct the study.

## Results

### Accuracies of Symptom Checkers

In this section, we present our findings of stage 3. As indicated in the Methods section, the 400 gold-standard vignettes were tested over 6 symptom checkers, namely Avey, Ada, WebMD, K Health, Buoy, and Babylon. Not every vignette was successfully diagnosed by every symptom checker. For instance, 18 vignettes failed on K Health because their constituent chief complaints were not available in K Health’s search engine; hence, the sessions could not be initiated. Moreover, 35 vignettes failed on K Health because of an age limitation (only vignettes that encompassed ages of ≥18 years were accepted by K Health).

In addition to search and age limitations, some symptom checkers (in particular, Buoy) crashed while diagnosing certain vignettes, even after trying multiple times. Moreover, many symptom checkers did not produce differential diagnoses for some vignettes albeit concluding the diagnostic sessions. For example, Babylon did not generate differential diagnoses for 351 vignettes. The reason some symptom checkers could not produce diagnoses for some vignettes is uncertain, but we conjecture that it might relate to either not modeling those diagnoses or falling short of recalling them despite being modeled. Table 3 summarizes the failure rates and reasons across the examined symptom checkers. Moreover, the table shows the average number of questions asked by each symptom checker upon successfully diagnosing vignettes.

**Table 3:** Failure reasons, failure counts, success counts, and average number of questions across the 6 tested symptom checkers.

Symptom checker	Failure reasons and counts				Success counts			Number of questions, mean (SD)
	Search limitations	Age limitations	Crashed	No DDx^a^ generated		DDx generated		
Avey	0	0	0	2		398	24.89 (12.15)	
Ada	0	0	0	0		400	29.33 (6.62)	
WebMD	2	1	0	3		394	2.64 (2.11)	
K Health	18	35	0	2		345	25.23 (6.59)	
Buoy	2	3	5	74		316	25.67 (5.79)	
Babylon	15	0	0	351		34	5.91 (5.47)	

^a^DDx: differential diagnosis.

**Figure 3:**
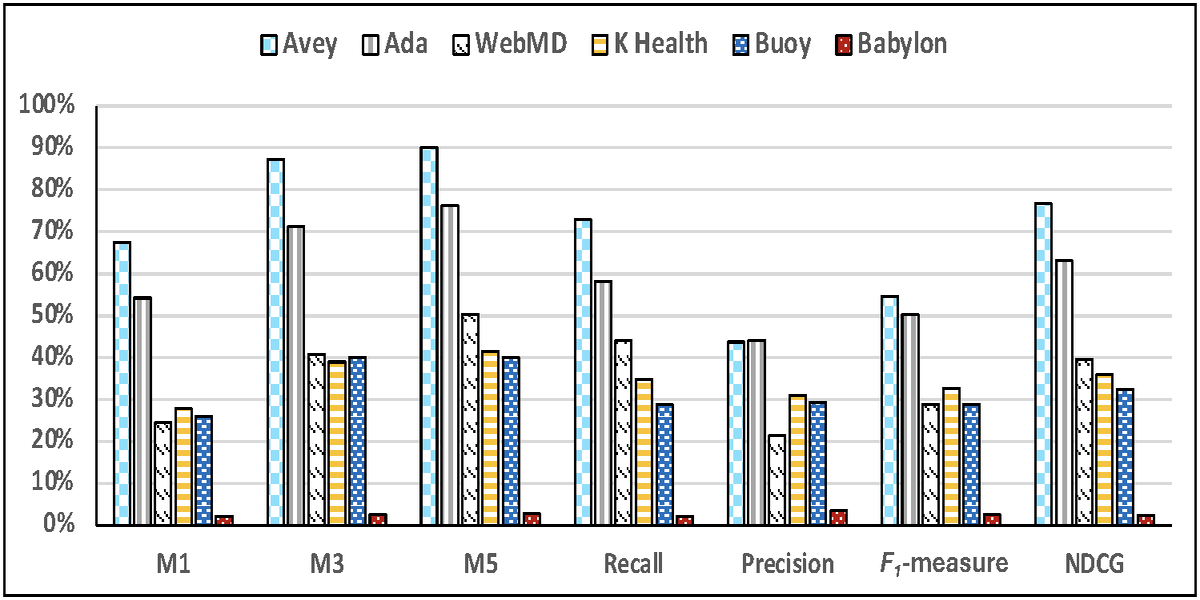
Accuracy results considering for each symptom checker all the succeeded and failed vignettes. NDCG: Normalized Discounted Cumulative Gain.

Figure 3 demonstrates the accuracy results of all the symptom checkers over the 400 vignettes, irrespective of whether they failed or not during some diagnostic sessions. In this set of results, a symptom checker is penalized if it fails to start a session, crashes, or does not produce a differential diagnosis albeit concluding the session. As depicted, Avey outperformed Ada, WebMD, K Health, Buoy, and Babylon, respectively, by averages of 24.5%, 175.5%, 142.8%, 159.6%, and 2968.1% using *M*1; 22.4%, 114.5%, 123.8%, 118.2%, and 3392% using *M*3; 18.1%, 79.2%, 116.8%, 125%, and 3114.2% using *M*5; 25.2%, 65.6%, 109.4%, 154%, and 3545% using recall; 8.7%, 88.9%, 66.4%, 88.9%, and 2084% using *F*_1_-measure; and 21.2%, 93.4%, 113.3%, 136.4%, and 3091.6% using NDCG. Ada was able to surpass Avey by an average of 0.9% using precision, although Avey outpaced it across all the remaining metrics, even with asking an average of 17.2% lesser number of questions (Table 3). As shown in Figure 3, Avey also outperformed WebMD, K Health, Buoy, and Babylon by averages of 103.2%, 40.9%, 49.6%, and 1148.5% using precision, respectively.

**Figure 4:**
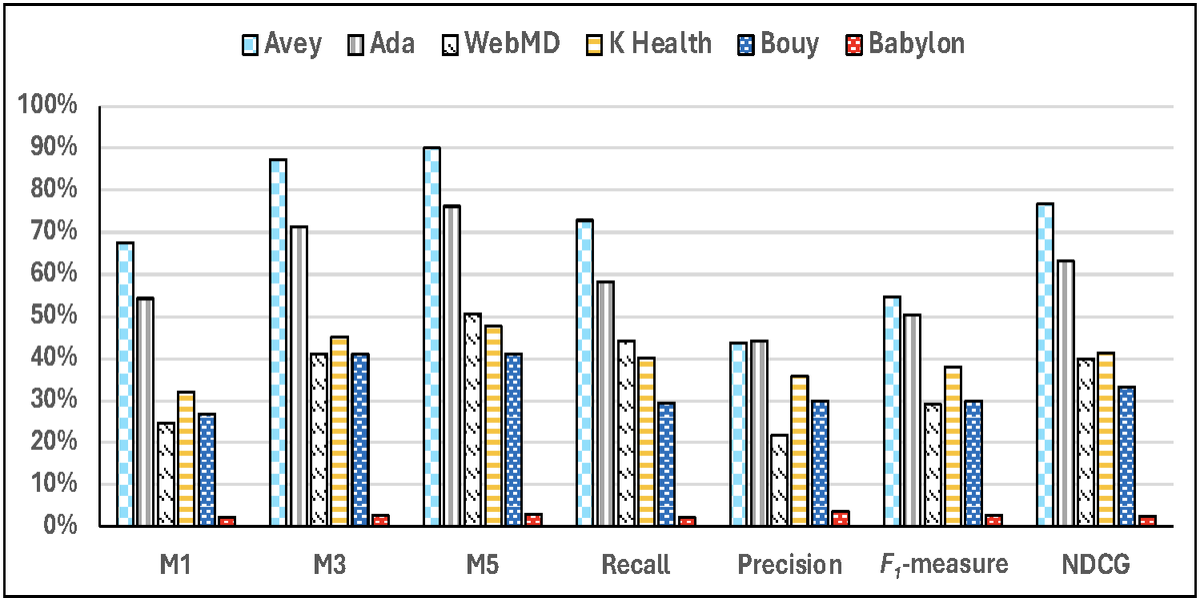
Accuracy results considering for each symptom checker only the succeeded vignettes, with or without differential diagnoses. NDCG: Normalized Discounted Cumulative Gain.

Figure 4 illustrates the accuracy results of all the symptom checkers across only the vignettes that were successful. In other words, symptom checkers were not penalized if they failed to start sessions or crashed during sessions. As shown in the figure, Avey outperformed Ada, WebMD, K Health, Buoy, and Babylon, respectively, by averages of 24.5%, 173.2%, 110.9%, 152.8%, and 2834.7% using *M*1; 22.4%, 112.4%, 94%, 112.9%, and 3257.6% using *M*3; 18.1%, 77.8%, 88.2%, 119.5%, and 3003.4% using *M*5; 25.2%, 64.5%, 81.8%, 147.1%, and 3371.4% using recall; 8.7%, 87.6%, 44.4%, 83.8%, and 1922.2% using *F*_1_-measure; and 21.2%, 91.9%, 85%, 130.7%, and 2964% using NDCG. Under average precision, Ada outpaced Avey by an average of 0.9%, whereas Avey surpassed WebMD, K Health, Buoy, and Babylon by averages of 101.3%, 22%, 45.6%, and 1113.8%, respectively.

**Figure 5:**
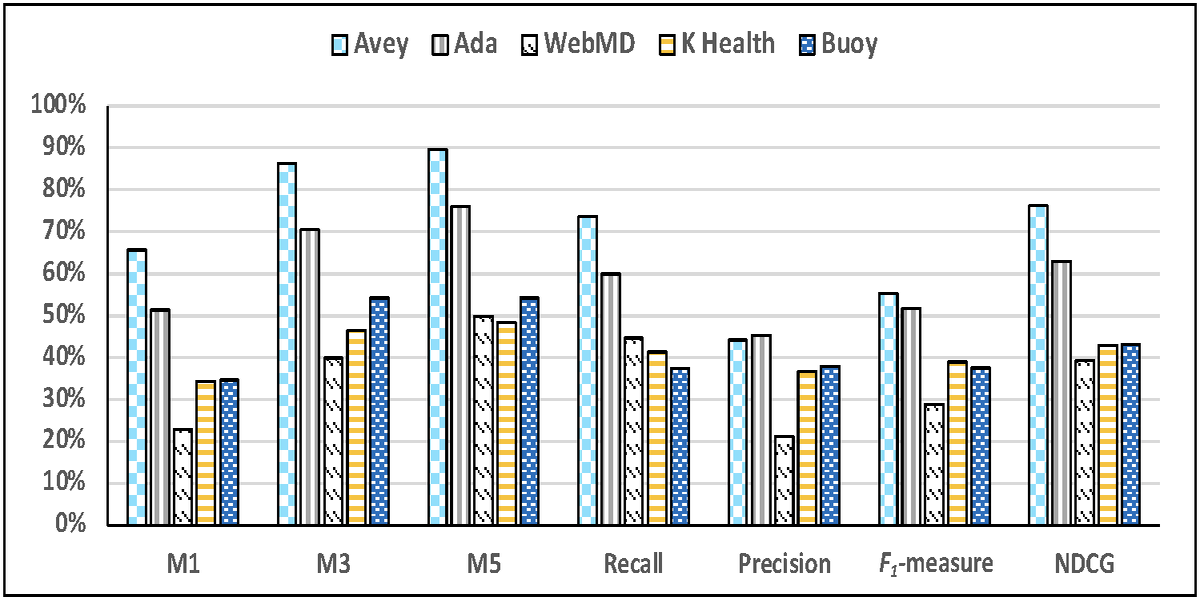
Accuracy results considering only the succeeded vignettes with differential diagnoses across all the symptom checkers. NDCG: Normalized Discounted Cumulative Gain.

Finally, Figure 5 shows the accuracy results of all the symptom checkers over only the vignettes that resulted in differential diagnoses on every symptom checker (ie, the intersection of successful vignettes with differential diagnoses across all symptom checkers). In this set of results, we excluded Babylon as it failed to produce differential diagnoses for 351 out of the 400 vignettes. As demonstrated in the figure, Avey outperformed Ada, WebMD, K Health, and Buoy, respectively, by averages of 28.1%, 186.9%, 91.5%, and 89.3% using *M*1; 22.4%, 116.3%, 85.6%, and 59.2% using *M*3; 18%, 80.1%, 85.7%, and 65.5% using *M*5; 23%, 64.9%, 78.5%, and 97.1% using recall; 7.2%, 92.7%, 42.2%, and 47.1% using *F*_1_-measure; and 21%, 93.6%, 77.4%, and 76.6% using NDCG. Under average precision, Ada surpassed Avey by an average of 2.4%, whereas Avey outpaced WebMD, K Health, and Buoy by averages of 109.5%, 20.4%, and 16.9%, respectively.

All the combinations of all the results (ie, 45 sets of experiments), including a breakdown between common and less-common diseases, are posted on the internet [27]. In general, we found Avey to be more accurate than the other 5 tested symptom checkers, irrespective of the combination of results; hence, it was chosen to be compared against primary care physicians.

### Avey Versus Human Doctors

In this section, we present our findings of stage 4. As discussed in the Methods section, we tested the 400 gold-standard vignettes on 3 doctors with an average clinical experience of 16.6 years. Table 4 shows the results of the doctors across all our accuracy metrics. Furthermore, Multimedia Appendix 2 depicts the results of Avey against the average physician, which is the average performance of the 3 physicians. As shown, the human doctors provided average *M*1, *M*3, *M*5, recall, precision, *F*_1_-measure, and NDCG of 61.2%, 72.5%, 72.9%, 46.6%, 69.5%, 55.3%, and 61.2%, respectively. In contrast, Avey demonstrated average *M*1, *M*3, *M*5, recall, precision, *F*_1_-measure, and NDCG of 67.5%, 87.3%, 90%, 72.9%, 43.7%, 54.6%, and 76.6%, respectively.

To this end, Avey compared favorably to the considered doctors, yielding inferior performance in terms of precision and *F*_1_-measure but a better performance in terms of *M*1, *M*3, *M*5, NDCG, and recall. More precisely, the doctors outperformed Avey by averages of 37.1% and 1.2% using precision and *F*_1_-measure, whereas Avey outpaced them by averages of 10.2%, 20.4%, 23.4%, 56.4%, and 25.1% using *M*1, *M*3, *M*5, recall, and NDCG, respectively.

**Table 4:** Accuracy results (%) of 3 medical doctors (MDs), MD_1_, MD_2_, and MD_3_, with an average experience of 16.6 years.

Doctors	M1	M3	M5	Recall	Precision	*F*_1_-measure	NDCG^a^
MD_1_	49.7	62	62.7	41.2	58.6	48.4	52.2
MD_2_	61.3	67.2	67.5	41.2	78.1	53.9	58
MD_3_	72.5	88.2	88.5	57.3	71.7	63.7	73.5

^a^NDCG: Normalized Discounted Cumulative Gain.

## Discussion

### Principal Findings

In this paper, we capitalized on the standard clinical vignette approach to assess the accuracies of 6 symptom checkers and 3 primary care physicians with an average experience of 16.6 years. We found that Avey is the most accurate among the considered symptom checkers and compares favorably to the 3 involved physicians. For instance, under *M*1, Avey outperforms the next best-performing symptom checker, namely, Ada, by 24.5% and the worst-performing symptom checker, namely Babylon, by 2968.2%. On average, Avey outperforms the 5 competing symptom checkers by 694.1% using *M*1. In contrast, under *M*1, Avey underperforms the best-performing physician by 6.9% and outperforms the worst-performing one by 35.8%. On average, Avey outperforms the 3 physicians by 13% using *M*1. Table 5 shows the ordering of symptoms and physicians from best-performing to worst-performing.

**Table 5:** Ordering of symptom checkers and physicians (denoted as MD_1_, MD_2_, and MD_3_) from best-performing to worst-performing symptom checkers and physicians.

Metrics	Descending order (best to worst)	Symptom checkers			Doctors	
		Values, range (%)	Values, SD (%)	Values, range (%)		Values, SD (%)
M1%	MD_3_, Avey, MD_2_, Ada, MD_1_, K Health, Buoy, WebMD, and Babylon	65.3	21	22.8		9
M3%	MD_3_, Avey, Ada, MD_2_, MD_1_, WebMD, Buoy, K Health, and Babylon	84.8	27	26.2		11
M5%	Avey, MD_3_, Ada, MD_2_, MD_1_, WebMD, K Health, Buoy, and Babylon	87.2	27	25.8		11
Average recall	Avey, Ada, MD_3_, WebMD, MD_1_ and MD_2_ (a tie), K Health, Buoy, and Babylon	70.9	22	16.1		8
Average precision	MD_3_, MD_2_, MD_1_, Ada, Avey, K Health, Buoy, WebMD, and Babylon	40.6	13	19.5		8
Average *F*_1_-measure	MD_3_, Avey, MD_2_, Ada, MD_1_, K Health, Buoy and WebMD (a tie), and Babylon	32.9	16	15.3		6
Average NDCG^a^	Avey, MD_3_, Ada, MD_2_, MD_1_, WebMD, K Health, Buoy, and Babylon	74.2	23	21.3		9

^a^NDCG: Normalized Discounted Cumulative Gain.

### Strengths and Limitations

This paper proposed a comprehensive and rigorous experimentation methodology that taps into the standard clinical vignette approach to evaluate symptom checkers and primary care physicians. On the basis of this methodology, we developed and peer reviewed the largest benchmark vignette suite in the domain thus far. A recent study used 200 vignettes and was deemed one of the most comprehensive to date [28]. The work of Semigran et al [29] used 45 vignettes and many studies followed suit [4,7,12,38].

Using this standardized suite, we evaluated the performance of a new AI symptom checker, namely, Avey; 5 popular symptom checkers, namely, Ada, WebMD, K Health, Buoy, and Babylon; and a panel of 3 experienced physicians to put things in perspective and interpret results accordingly. To measure accuracy, we used 7 standard metrics, one of which was leveraged for the first time in literature to quantify the ranking qualities of symptom checkers’ and physicians’ differential diagnoses. To minimize bias, the 6 symptom checkers were tested by only independent primary care physicians and using only peer-reviewed vignettes.

To facilitate the reproducibility of the study and support future related studies, we made all the peer-reviewed vignettes publicly and freely available on the internet [27]. In addition, we posted on the internet all the reported results (eg, the screenshots of the sessions with symptom checkers and the answers of physicians) on the Benchmark Vignette Suite [27] to establish a standard of full transparency and allow for external cross-validation.

That said, this study lacks an evaluation with real patients and covers only 14 body systems with a limited range of conditions. As pointed out in the Methods section, in the clinical vignette approach, vignettes act as proxies for real patients. The first step in this approach is to standardize these vignettes, which would necessitate an assembly of independent and experienced physicians to review and approve them. Consequently, if we replace vignettes with real patients, a group of physicians (say, 7, as is the case in this study, for example) is needed to check each patient at the same time and agree by a supermajority vote on their differential diagnosis. This corresponds to standardizing the diagnosis of the patient before she or he is asked to self-diagnose with each symptom checker. Afterward, the diagnoses of the symptom checkers can be matched against the patient’s standardized diagnosis and accuracy results can be reported accordingly.

Albeit appealing, the abovementioned approach differs from the standard clinical vignette approach (wherein no vignettes will be involved anymore but actual patients) and is arguably less practical, especially since it suggests checking and diagnosing a vast number of patients, each by a panel of physicians, before testing on symptom checkers. In addition, the cases of the patients should cover enough diseases (eg, as in Table 1), which could drastically increase the pool of patients that needs to be diagnosed by physicians before identifying a representative sample. This may explain why this alternative approach has not been used in any of the accuracy studies of symptom checkers so far, granted that the clinical vignette approach is a standard paradigm, let alone that it is also commonly used for testing the diagnostic abilities of physicians [29].

In any of these approaches, it is important to distinguish between *testers* and *subjects*. For instance, in the abovementioned alternative approach, the patients are the testers of the symptom checkers and the subjects by which the symptom checkers are tested. In contrast, in the clinical vignette approach, the testers are either physicians or laypeople, whereas the subjects are the standardized vignettes. As discussed in the Stage 3: Vignette Testing on Symptom Checkers section, using physicians as testers makes the clinical vignette approach more reliable. This is because symptom checkers may ask questions that hold no answers in the standardized vignettes, making it difficult for laypeople to answer them appropriately and hard for the community to trust the reported results accordingly.

To this end, 2 research methodologies have been adopted in the literature. One is to dry run a priori by a physician every gold-standard vignette on every considered symptom checker and identify every finding (ie, symptom, etiology, or attribute) that could be asked by these symptom checkers. Subsequently, the physician supplements each vignette with more findings to ensure that laypeople can properly answer any question asked during actual testing. This is the methodology that was used in the seminal work of Semigran et al [11,29].

The second methodology is not to dry run each vignette beforehand on each symptom checker, especially as it might not be possible to fully know what an AI-based symptom checker will ask during actual testing. On the contrary, the methodology suggests standardizing the vignettes in a way that precisely reflects real-life patient cases. Afterward, multiple (to address bias and ensure reliability) independent physicians test the vignettes on each symptom checker. These physicians will then reliably answer any questions about any data not included in the vignettes, thus ensuring the correctness of the approach. This methodology has been shown to be more reliable for conducting accuracy studies [28,38,47]. As such, it was used in most recent state-of-the-art papers [4,28] and, consequently, in ours.

Aside from studying the accuracy of symptom checkers, real patients can be involved in testing the usability of such tools (eg, by using a self-completed questionnaire after self-diagnosing with symptom checkers as in the study by Miller et al [55]). Clearly, this type of study is orthogonal to the accuracy ones and lies outside the scope of this paper.

Finally, we indicate that the physicians that were compared against the symptom checkers in stage 4 (ie, vignette testing on doctors) may not be a representative sample of primary care physicians. Furthermore, our study did not follow a rigorous process to choose symptom checkers and considered only a few of them, which were either new (ie, Avey), popular (ie, Babylon), or performed superiorly in recent studies (ie, Ada, K Health, Buoy, and WebMD).

### Comparison With the Wider Literature

Much work, especially recently, has been done to study symptom checkers from different perspectives. It is not possible to do justice to this large body of work in this short paper. As such, we briefly describe some of the most closely related ones, which focus primarily on the accuracy of self-diagnosis.

Semigran et al [29] were the first to study the performance of many symptom checkers across a range of conditions in 2015. They tested 45 vignettes over 23 symptom checkers and discovered that their accuracies vary considerably, with *M*1 ranging from 5% to 50% and *M*20 (which measures if a symptom checker returns the gold-standard main diagnosis among its top 20 suggested conditions) ranging from 34% to 84%.

Semigran et al [11] published a follow-up paper in 2016 that compared the diagnostic accuracies of physicians against symptom checkers using the same vignettes in Semigran et al [29]. Results showed that, on average, physicians outperformed symptom checkers (72.1% vs 34.0% along *M*1 and 84.3% vs 51.2% along *M*3). However, symptom checkers were more likely to output the gold-standard main diagnosis at the top of their differentials for low-acuity and common vignettes, whereas physicians were more likely to do so for high-acuity and uncommon vignettes.

The 2 studies of Semigran et al [11,29] provided useful insights into the first generation of symptom checkers. However, much has changed from 2015 to 2016. To exemplify, Gilbert et al [28] recently compiled, peer reviewed, and tested 200 vignettes over 8 popular symptom checkers and 7 primary care physicians. As in the study by Semigran et al [29], they found a significant variance in the performance of symptom checkers, but a promise in the accuracy of a new symptom checker named Ada [41]. Ada exhibited accuracies of 49%, 70.5%, and 78% under *M*1, *M*3, and *M*5, respectively.

None of the symptom checkers in the study by Gilbert et al [28] outperformed general practitioners but Ada came close, especially in *M*3 and *M*5. The authors of the study by Gilbert et al [28] pointed out that the nature of iterative improvements in software suggests an expected increase in the future performance of symptom checkers, which may at a point in time exceed that of general practitioners. As illustrated in Figure 2, we found that Ada is still largely ahead of the conventional symptom checkers but Avey outperforms it. Furthermore, Avey surpassed a panel of physicians under various accuracy metrics as depicted in Multimedia Appendix 2.

Hill et al [4] evaluated 36 symptom checkers, 8 of which use AI, over 48 vignettes. They showed that accuracy varies considerably across symptom checkers, ranging from 12% to 61% using *M*1 and from 30% to 81% using *M*10 (where the correct diagnosis appears among the top 10 conditions). They also observed that AI-based symptom checkers outperform rule-based ones (ie, symptom checkers that do not use AI). Akin to Hill et al [4], Ceney et al [12] detected a significant variation in accuracy across 12 symptom checkers, ranging from 22.2% (Caidr [56]) to 72% (Ada) using *M*5.

Many other studies focused on the diagnostic performance of symptom checkers, but only across a limited set of diagnoses [57-68]. For instance, Shen et al [67] evaluated the accuracy of WebMD for ophthalmic diagnoses. Hennemann et al [62] investigated the diagnostic performance of Ada for mental disorders. Nateqi et al [65] validated the accuracies of Symptoma [69], Ada, FindZebra [70], Mediktor [71], Babylon, and Isabel [72] for ear, nose, and throat conditions. Finally, Munsch et al [64] assessed the accuracies of 10 web-based COVID-19 symptom checkers.

From a technical perspective, early AI models for medical diagnosis adopted expert systems [49,73-76]. Subsequent models used probabilistic formulations to account for uncertainty in the diagnostic process [77] and focused on approximate probabilistic inference to optimize for efficiency [78-80].

With the increasing availability of electronic medical records (EMRs), Rotmensch et al [81] used logistic regression, naive Bayes, and Bayesian networks with noisy OR gates (noisy OR) on EMRs to automatically construct medical knowledge graphs. Miotto et al [82] proposed an EMR-based unsupervised deep learning approach to derive a general-purpose patient representation and facilitate clinical predictive modeling. Ling et al [83] modeled the problem as a sequential decision-making process using deep reinforcement learning. Kannan et al [49] showed that multiclass logistic regression and deep learning models can be effective in generalizing to new patient cases, but with an accuracy caveat concerning the number of diseases that can be incorporated.

Miller et al [55] presented a real-world usability study of Ada over 523 participants (patients) in a South London primary care clinic over a period of 3 months. Approximately all patients (ie, 97.8%) found Ada very easy to use. In addition, 22% of patients aged between 18 and 24 years suggested that using Ada before coming to the clinic would have changed their minds in terms of what care to consider next. Studies of other symptom checkers such as Buoy and Isabel reported high degrees of utility as well [24,84].

Some other work has also explored the triage capabilities of symptom checkers [7,38,84-86]. Studying the utility and triage capabilities of symptom checkers is beyond the scope of this paper and has been set as future work in the Unanswered Questions and Future Research section.

Finally, we note that many survey papers systematically reviewed symptom checkers, made several observations, and identified a few gaps [12,20,23,53,86-91]. For instance, Chambers et al [87] found in 2019 that symptom checkers were much less accurate than physicians. This was observed in this study as well for most of the symptom checkers (see the Results section). Aboueid et al [12] identified knowledge gaps in the literature and recommended producing more research in this area with a focus on accuracy, user experience, regulation, doctor-patient relationship, primary care provider perspectives, and ethics. Finally, some studies [88-90] highlighted various challenges and opportunities in using symptom checkers. They revealed methodological variability in triage and diagnostic accuracies and, thus, urged for more rigorous and standardized evaluations before widespread adoption. In response to this, our work used the standard clinical vignette approach to study the diagnostic accuracies of some commonly used symptom checkers.

### Implications for Clinicians and Policy Makers

As pointed out in the Introduction section, a United Kingdom–based study that engaged 1071 patients found that >70% of individuals aged between 18 and 39 years would use a symptom checker [13]. This study was influential in the United Kingdom health policy circles, whereby it received press attention and prompted responses from National Health Service England and National Health Service X, a United Kingdom government policy unit that develops best practices and national policies for technology in health [55,92]. Given that symptom checkers vary considerably in performance (as demonstrated in the Results section), this paper serves to scientifically inform patients, clinicians, and policy makers about the current accuracies of some of these symptom checkers.

Finally, this study suggests that any external scientific validation of any AI-based medical diagnostic algorithm should be fully transparent and eligible for replication. As a direct translation to this suggestion, we posted all the results of the tested symptom checkers and physicians on the web to allow for cross-verification and study replication. Moreover, we made all peer-reviewed vignettes in our study publicly and freely available. This will not only enable the reproducibility of our study but also further support future related studies, both in academia and industry alike.

### Unanswered Questions and Future Research

This paper focused solely on studying the diagnostic accuracies of symptom checkers. Consequently, we set forth 2 complementary future directions, namely, usability and utility. To elaborate, we will first study the usability and acceptability of symptom checkers with real patients. In particular, we will investigate how patients will perceive symptom checkers and interact with them. During this study, we will observe and identify any barrier in the user experience or user interface and language characteristics of such symptom checkers. Finally, we will examine how patients will respond to the output of these symptom checkers and gauge their influence on their subsequent choices for care, especially when it comes to triaging.

### Conclusions

In this paper, we proposed an experimentation methodology that taps into the standard clinical vignette approach to evaluate and analyze 6 symptom checkers. To put things in perspective, we further compared the symptom checker that demonstrated the highest performance, namely, Avey against a panel of experienced primary care physicians. Results showed that Avey outperforms the 5 other considered symptom checkers, namely, Ada, K Health, Buoy, Babylon, and WebMD by a large margin and compares favorably to the participating physicians. The nature of iterative improvements in software and the fast pace of advancements in AI suggest an accelerated increase in the future performance of such symptom checkers.

## Appendix

Multimedia Appendix 1 The alignment of our methodology with the recommended requirements of pursuing the clinical vignette approach.

Multimedia Appendix 2 Accuracy results of Avey versus 3 medical doctors (MDs), on average (ie, average MD). NDCG: Normalized Discounted Cumulative Gain.

## Abbreviations

AI: artificial intelligence
DCG: Discounted Cumulative Gain
EMR: electronic medical record
NDCG: Normalized Discounted Cumulative Gain

## Data Availability

All our gold-standard vignettes are made publicly and freely available [93] to enable the reproducibility of this work. In addition, all the outputs of the symptom checkers and physicians are posted at the same site to allow for external cross-validation. Finally, the results of all our 45 sets of experiments are published [94] to establish a standard of full transparency.
