# Supplementary material for: Evaluating the Diagnostic Performance of Symptom Checkers: Clinical Vignette Study
Source: JMIR AI. 2024 Apr 29;3:e46875. doi: 10.2196/46875 (PMC11091811; doi:10.2196/46875)
Supplement: Multimedia Appendix 1 [file ai_v3i1e46875_app1.docx]

**Appendix A: The alignment of our methodology with the recommended requirements of pursuing the clinical vignette approach.**

| Category | Recommendations from [82] | Reference or Justification |
| --- | --- | --- |
| Vignettes | Illustrate the method for determining the minimum number of vignettes required for Online Symptom Checker (OSC) evaluation | See section “Strengths and Limitations”, paragraph 1. Our paper compiled, peer-reviewed (for standardization purposes), and used for testing symptom checkers the largest vignette suite in the domain so far, which encompassed 400 vignettes. To compare and contrast, a recent study utilized 200 vignettes and was deemed one of the most comprehensive to date [33]. The seminal work of [34] utilized 45 vignettes and many studies followed suit [4, 7, 12, 43]. |
|  | Specify the minimum information to be included in each vignette | See section “Stage 1: Vignette Creation Stage”, paragraph 2. The minimum information to be included in each vignette is the least amount of information needed to accurately identify the differential diagnosis in it. To this end, each vignette in our suite consisted of eight major components: (i) the age and sex of the assumed patient, (ii) a maximum of three chief complaints, (iii) the history of the suggested illness associated with details on the chief complaints and other present and relevant findings (a finding is defined as a symptom, a sign, or an etiology, each with a potential attribute), (iv) absent findings, including ones that are expected to be solicited by symptom checkers and physicians in stages 3 and 4, (v) basic findings that pertain to physical examinations that can still be exploited by symptom checkers, (vi) past medical and surgical history, (vii) family history, and (viii) the most appropriate main and differential diagnoses. We made all our peer-reviewed vignetted open-source at [84]. |
|  | Provide guidance on determining the conditions, symptoms, or spread of cases to be included and assurance that this appropriately represents the target user population | See section “Stage 1: Vignette Creation Stage”. The conditions and the spread of the symptoms were determined based on how commonly they occur with general public patients (our target user population). More precisely, 222 vignettes (or 55.5% of the total number vignettes used in the paper) encompass wide-spread, common diseases (see Table 1) drawn from reliable sources that depend on practical clinical practice [35, 36, 37, 38, 39, 40, 41, 42]. To exemplify, we included pulmonology conditions, which vary from most common (e.g., flu, common cold, and COVID-19) to less common (e.g., tuberculosis and lung cancer).    To expand representation and coverage, we further included 178 vignettes presenting less common diseases (e.g., lyme disease or dengue fever) that are also experienced by general public patients. We open-sourced all the vignettes (which comprise all the diseases and symptoms) at [84] to establish a standard of full transparency and allow for external cross-validation. |
|  | Specify vignette origin requirements (e.g., simulated vs real-world cases) | See section “Stage 1: Vignette Creation Stage”, paragraph 1. The vignettes represent real-world cases and were all drawn from reputable sources. |
| Clinician assessment | Specify the appropriate clinicians (including role, specialty, and seniority) to be used in the assignment of ‘gold standard’ labels | **Stage 1: Vignette Creation Stage**  Dr. Sara Alawneh, Family medicine, 3 years experience, Doha - Qatar  Dr. Duaa Ahmad, General medicine, 3 years experience, Doha - Qatar  Dr. Azmi Qudsi, General medicine, 3 years experience, Amman - Jordan  **Stage 2: Vignette Standardization Stage**  Dr. Arwa Khashan, Family medicine, 20 years experience, Amman - Jordan  Dr. Odai Albatsh, Emergency medicine, 7 years experience, Jeddah - KSA  Dr. Ahmad Alowaidat, Emergency medicine, 6 years experience, Amman - Jordan  Dr. Zaid Abo Saleh, Emergency medicine, 5 years experience, Amman - Jordan  Dr. Nour Essale, General medicine, 6 years experience, United Kingdom  Dr. Muna Darmach, Internal medicine, 7 years experience, Amman - Jordan  Dr. Tamara ALtawara, Family medicine, 8 years experience, Amman - Jordan  **Stage 3: Vignette Testing on Symptom Checkers**  Dr. Muna Darmach, Internal medicine, 7 years experience, Amman - Jordan  Dr. Ahmed Kakakhan, General medicine, 2 experience, Amman - Jordan  Dr. Maram Smairat, Family medicine, 3.5 years experience, Amman - Jordan  **Stage 4: Vignette Testing on Doctors**  Dr. Noor Jodeh, Family medicine, 10 years experience, Amman - Jordan  Dr. Tala Hamouri, Family medicine, 10 years experience, Amman - Jordan  Dr. Mohmmad Almadani, Family medicine, 30 years experience, Amman - Jordan |
|  | Illustrate the method used for compilation of a gold standard (e.g., averaged single blinded assessment or consensus discussion) | The vignettes were compiled in stage 1 (i.e., vignette creation stage) using a consensus discussion and peer-reviewed in stage 2 (i.e., vignette standardization stage) using a single-blinded assessment. Physicians were unable to see the reviews of other physicians to avoid bias. |
| Triage | Specify standardized triage categories, including setting and time periods | Not applicable (our study focuses solely on diagnostic accuracy- see section “Comparison to the Wider Literature”, paragraph 11). |
|  | Provide guidance on the use of triage gold standards as a range for both urgency and setting | Not applicable (our study focuses solely on diagnostic accuracy- see section “Comparison to the Wider Literature”, paragraph 11). |
| Differential diagnosis | Illustrate the method for comparing OSC differential diagnosis list to gold standard dispositions | We compared the gold-standard differential diagnoses against symptom checkers’ diagnoses using 7 methods, namely, M1, M3, M5, Recall, Precision, F1-Score, and NDCG (see Table 2). For instance, we did not only compare whether a symptom checker would return the diseases within a gold-standard differential list (captured through the Recall metric), but also whether it would return them in *the right order* (captured through the NDCG metric). To the best of our knowledge, our study is the first in the field to pursue this approach and quantify the qualities of differential rankings as generated by symptom checkers. |
| Accuracy | Illustrate the accuracy and safety score calculation method, accounting for outcomes that fall both below and above the gold standard | See Table 2. |
|  | Specify the minimum accepted accuracy and safety scores | See Figures 3, 4, and 5. See also Table 5. |
| Safety netting | Specify how safety netting contributes to product safety scores | Not applicable (our study focuses solely on diagnostic accuracy). |
| Inputters | Specify the minimum interrater reliability scores for inputters of vignettes | See section “Stage 3: Vignette Testing on Symptom Checkers”. Three independent primary care physicians under two specialties, namely, Family Medicine and General Medicine, with an average experience of 4.2 years tested the gold-standard vignettes on the six considered symptom checkers. To avoid bias, each physician *randomly* pulled vignettes from the gold-standard pool and tested them on each of the six symptom checkers. |
|  | Illustrate the method for determining the number of tests and inputters required for each vignette | See section “Stage 3: Vignette Testing on Symptom Checkers”. By the end of stage 3, each physician tested a total of 133 gold-standard vignettes on each symptom checker, except one physician who tested one extra vignette to exhaust the 400 vignettes (the full gold-standard vignette suite). This is because each vignette was tested by one physician on the six considered symptom checkers. Each physician saved a screenshot of each symptom checker’s output for each vignette to facilitate results’ verification. All the screenshots were posted online at [31] to establish a standard of full transparency and allow for external cross-validation and study-replication. |
|  | Specify the appropriate characteristics for vignette inputters (e.g., medical education level and affiliation with developers) | See section “Stage 3: Vignette Testing on Symptom Checkers”, paragraph 1. None of the inputters had any involvement with the development of any of the symptom checkers tested in this study. As shown in the “clinical assessment” row above (see “Stage 3: Vignette Testing on Symptom Checkers”), all the inputters are experienced practicing physicians. |
