# Supplementary figures and images for: Evaluating the Diagnostic Performance of Symptom Checkers: Clinical Vignette Study
Source: JMIR AI. 2024 Apr 29;3:e46875. doi: 10.2196/46875 (PMC11091811; doi:10.2196/46875)

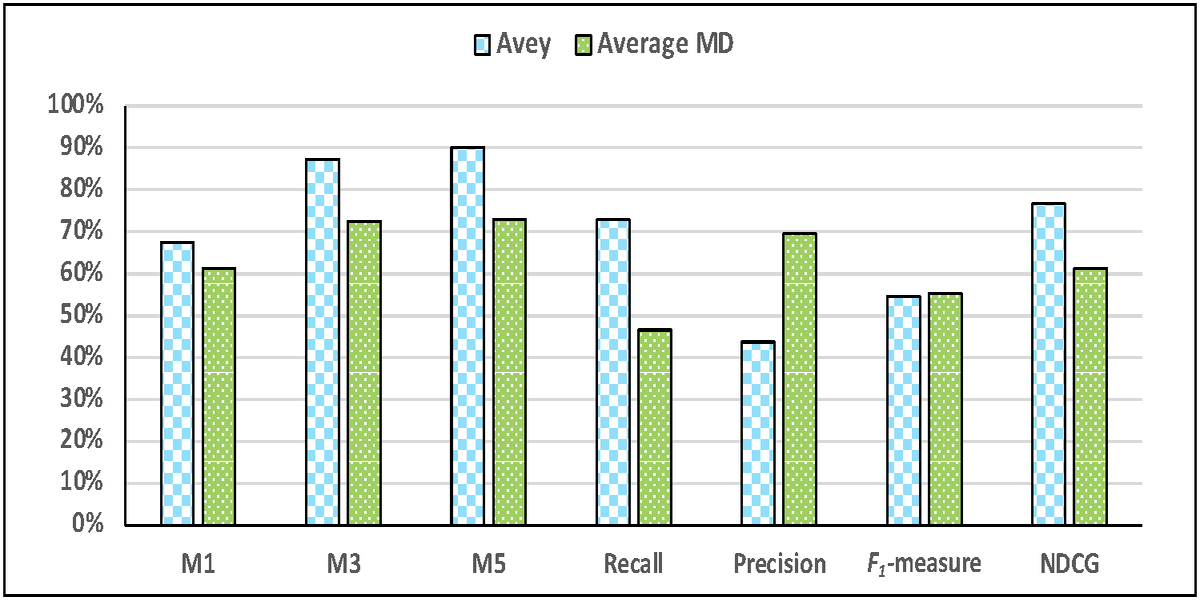

Supplement: Multimedia Appendix 2 [file ai_v3i1e46875_app2.png]
